# Feasibility and safety of robotic surgery for low rectal cancer combined with transanal total mesorectal excision

**DOI:** 10.1007/s00423-023-02870-z

**Published:** 2023-03-29

**Authors:** Masayuki Ando, Takeru Matsuda, Ryuichiro Sawada, Hiroshi Hasegawa, Kimihiro Yamashita, Hitoshi Harada, Naoki Urakawa, Hironobu Goto, Shingo Kanaji, Taro Oshikiri, Yoshihiro Kakeji

**Affiliations:** 1 Division of Gastrointestinal Surgery, Department of Surgery, Kobe University Graduate School of Medicine, Kobe, Japan; 2 Division of Minimally Invasive Surgery, Department of Surgery, Kobe University Graduate School of Medicine, 7-5-2 Kusunoki-Chou, Chuo-Ku, Kobe, 650-0017 Japan

**Keywords:** TaTME, Robotic surgery, Hybrid surgery, Rectal cancer, Propensity score matching

## Abstract

**Purpose:**

Laparoscopic surgery for low rectal cancer is often challenging. Transanal total mesorectal excision (TaTME) and robotic surgery have been introduced to overcome the technical difficulties in laparoscopic surgery and achieve more favorable outcomes. Hybrid robotic surgery, which combines TaTME with the abdominal robotic approach, incorporates the advantages of each of these surgical techniques and might achieve less invasive and safer surgery. This study evaluated the safety and feasibility of hybrid robotic surgery with TaTME (hybrid TaTME).

**Methods:**

We retrospectively reviewed 162 TaTME cases performed at our department from September 2016 to May 2022. Among them, 92 cases of conventional TaTME and 30 of hybrid TaTME were eligible. We used propensity score matching analysis (PSM) to adjust for patients’ characteristics and compared the short-term outcomes of the two treatment groups.

**Results:**

Twenty-seven cases in each group were extracted using PSM. The operation time in hybrid TaTME was comparable to that in conventional TaTME. There was no significant difference in the postoperative hospital stay between the two groups. Other intra- and post-operative outcomes were also comparable between the two groups. Furthermore, no significant differences were observed between the two groups in the curative resection and recurrence rates.

**Conclusion:**

Hybrid TaTME for low rectal cancer was as favorable as conventional TaTME in producing satisfactory short-term outcomes. However, furthermore, larger-scale studies conducted over longer study periods are needed to evaluate the validity of the findings.

## Introduction

Total mesorectal excision (TME), which was first documented by Heald in 1982, has become the standard surgical technique for rectal cancer [1, 2]. Complete TME and a secure circumferential resection margin (CRM) have been considered very important in and have contributed to reduced local recurrence [3–6]. Although laparoscopic surgery for rectal cancer was introduced, complete TME has been challenging in cases such as patients with obesity and male patients with a narrow pelvis [7–9]. Although two large randomized control trials (RCTs) had found that laparoscopic surgery was not inferior to open surgery in oncological outcomes, two large subsequent RCTs refuted that laparoscopic surgery was not non-inferior to open surgery in terms of securing CRM, which thus raised concerns regarding laparoscopic surgery [10–13]. Robotic surgery and transanal TME (TaTME) have recently emerged as independent attempts to overcome these technical difficulties.

Robotic surgery provides three-dimensional vision, articulated function, and image stabilization, enabling safer and more meticulous manipulation in the complex pelvis [14, 15]. In the large-scale ROLARR trial, no statistically significant differences were found between robotic-assisted laparoscopic surgery and laparoscopic surgery in the overall conversion rate, positive CRM, and perioperative complications [15]. However, the conversion rate in robotic-assisted surgery was significantly lower in male patients, patients with obesity, and patients who underwent low anterior resection [15]. These results suggest that robotic surgery may be indicated for cases that are difficult to treat with laparoscopic surgery.

TaTME is a transanal TME procedure performed in a down-to-up fashion. TaTME provides a better view in lower rectal cancer and secures the distal margin (DM), leading to better oncological outcomes [16, 17]. However, issues have been raised concerning pelvic organ injuries, such as urethral injury, and a high local recurrence rate in TaTME because of technical difficulties; thus, technical proficiency is required in TaTME [17–20].

We have performed TaTME in more than 160 cases since its first introduction in September 2016. TaTME is currently the first-line surgical procedure for low rectal cancer at our department. Since June 2020, we have performed TaTME in combination with the abdominal robotic approach (hybrid TaTME) for a safer, less invasive surgery. Because reports on this technique are scarce, the feasibility and safety of hybrid TaTME are unclear [21–24]. Therefore, this study evaluated the feasibility and safety of hybrid TaTME compared with conventional TaTME for low rectal cancer.

## Materials and methods

### Patients

A database of prospectively managed rectal cancer cases at Kobe University was reviewed. We retrospectively examined 162 TaTME cases performed by our department from September 2016 to May 2022. Patients aged 20 years or older with preoperatively histologically proven malignant tumors, such as adenocarcinoma, squamous cell carcinoma, neuroendocrine tumor, or malignant melanoma, located below the peritoneal reflection, were eligible for this study. Five cases of robotic TaTME (in which robot-assisted anal manipulation was performed), 4 of total pelvic exenteration, 6 of inguinal lymph node dissection, 6 of total colectomy, 2 of perineal reconstruction, and 1 of laparotomy were excluded. Two patients diagnosed with gastrointestinal stromal tumors, 2 with benign tumors, 2 with double cancers, and 10 with preoperative recurrence were also excluded. In total, 92 cases of conventional laparoscopic TaTME and 30 of hybrid TaTME were included in the study. Patient characteristics; surgical, postoperative, and pathological outcomes; and recurrence data were extracted from electronic medical records in this study.

As described above, we introduced TaTME for low rectal cancer in 2016, and all patients with low rectal cancer were indicated for TaTME. Hybrid TaTME was introduced in June 2020. However, the availability of robot was limited in our institution, and hybrid TaTME was performed depending on its availability.

This study was approved by the Institutional Review Board and Ethics Committee of Kobe University Graduate School of Medicine (IRB reference no.: B220001).

### Preoperative management

All patients were diagnosed based on imaging studies, including colonoscopy, computed tomography, magnetic resonance imaging, and positron emission computed tomography. Patients diagnosed as cT3/4- or cN-positive without distant metastasis were eligible for preoperative treatment. Preoperative treatment consisted of neoadjuvant chemoradiotherapy (NACRT) using oral 5-FU and radiation therapy (45–50 Gy) or neoadjuvant chemotherapy (NAC). Surgery was performed 6–8 weeks after the completion of NACRT. The patients who received NAC underwent surgery 2–8 weeks after 6 courses of FOLFOXIRI and bevacizumab.

### Surgical techniques

Two teams performed the TaTME with simultaneous abdominal and perineal manipulation, except for the first six cases of conventional TaTME and the first of hybrid TaTME. The surgery was performed according to the procedure described by Lacy et al. [16].

For perineal manipulation in low anterior resection, the GelPOINT path (Applied Medical, USA) was applied through the anus, and CO_2_ was insufflated using an AirSeal (SurgiQuest, USA) to achieve a pressure of 13 mmHg. After confirming the DM of the tumor, the rectum was closed by applying a purse-string suture to the submucosa to secure a sufficient DM. The rectum was washed to prevent the contamination of the surgical field and tumor implantation. Then, the perineal team initiated a full-thickness rectal incision and performed TaTME in a down-to-up fashion, as described previously [25].

Perineal manipulation in abdominoperineal resection was performed as described previously [26]. Briefly, the anus was tightly closed by a double purse-string suture, and the skin was incised circumferentially around the anus. The GelPOINT Mini (Applied Medical, USA) was placed subcutaneously, and the perineal subcutaneous cavity was insufflated by CO_2_ at a pressure of 12–15 mmHg. The levator ani muscle was widely exposed and its transection was started at 6 o’clock. After exposure of the mesorectal plane, the dissection of the levator ani muscle was extended bilaterally. Then, the puborectal sling was dissected, and the dorsal surface of the prostate (or the vagina) was identified by dissecting between the neurovascular bundle and mesorectum. The rectourethral muscle (or the rectovaginal septum) was divided using the prostate (or the vagina) as a landmark. Finally, TME was performed in a down-to-up manner.

In the conventional surgery, the standard abdominal manipulations, including the ligation of the inferior mesenteric artery and transabdominal TME, were performed using a conventional 5-port system in parallel. In the hybrid surgery, the same surgical procedures as above were performed using the da Vinci Xi Surgical System (Intuitive Surgical, Sunnyvale, CA, USA).

When both the abdominal and perineal teams reached the peritoneal reflection, the peritoneum was incised, and the TME was completed. Then, the specimen was extracted through a mini-laparotomy. After the perineal team performed purse-string suturing on the distal rectal stump, they created an anastomosis using the single stapling technique. After confirming the presence of anastomotic bleeding and anastomotic leakage by colonoscopy, a drain was placed on the pelvic floor. A diverting ileostomy was subsequently created.

**Fig. 1:**
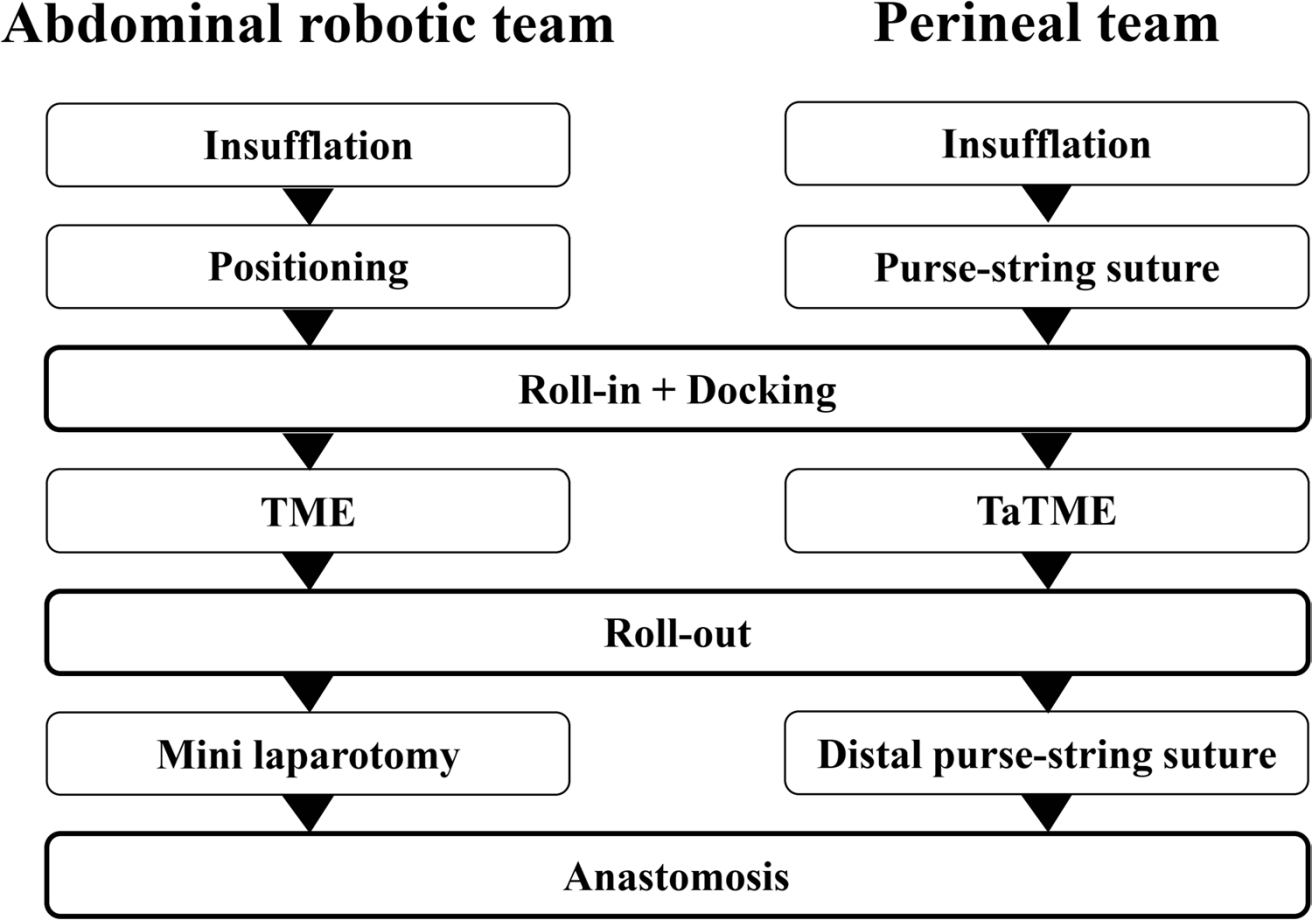
Diagram of the two-team process for hybrid transanal total mesorectal excision

In the hybrid surgery, the entire process was performed as described in Fig. 1.

### Statistical analysis

Propensity score matching analysis (PSM) was performed to reduce confounding bias between the conventional and hybrid TaTME groups. A propensity score was calculated by logistic regression analysis using age, sex, American Society of Anesthesiology score, body mass index, distance from the anal verge, clinical T stage, surgical procedure, preoperative treatment, and lateral pelvic lymph node dissection (LLND) as covariates. Nearest neighbor matching was performed so that the two groups were at a 1:1 ratio without replacement, and the width of the caliper was within 0.2 standard deviation (SD). Then, 27 patients each from the hybrid and conventional TaTME groups were matched.

Categorical variables were compared using the chi-square test or Fisher’s exact test. Non-parametric continuous variables were compared using the Mann–Whitney test and expressed as median and range. Local recurrence-free survival rate (LRFS) and recurrence-free survival rate (RFS) were calculated using the Kaplan–Meier method, and the log-rank test was performed to compare the survival curves of the two treatment groups. The results were considered statistically significant when the *P*-value < 0.05. All statistical data were analyzed using EZR version 1.54 (Saitama Medical Center, Jichi Medical University, Saitama, Japan), which is a graphical user interface for R (The R Foundation for Statistical Computing, Vienna, Austria).

## Results

Patient backgrounds and tumor characteristics are summarized in Table 1. The patients in the hybrid group were younger than those in the conventional group. The distance from the anal verge was shorter in the hybrid group than in the conventional group. The patient and tumor characteristics after PSM were similar between the two groups.

**Table1:** Patient and tumor characteristics before and after PSM

	Before PSM			After PSM		
	Conventional	Hybrid	*P*	Conventional	Hybrid	*P*
	*n* = 92	*n* = 30		*n* = 27	*n* = 27	
Age *	69.00 (33.00, 88.00)	58.00 (46.00, 86.00)	0.002	63.00 (38.00, 82.00)	58.00 (46.00, 86.00)	0.391
Sex, n (%)			0.385			1
Male	61 (66.3)	17 (56.7)		16 (59.3)	16 (59.3)	
Female	31 (33.7)	13 (43.3)		11 (40.7)	11 (40.7)	
BMI (kg/m^2^) *	23.00 (15.00, 37.00)	23.00 (19.00, 28.00)	0.804	23.00 (17.00, 28.00)	23.00 (19.00, 28.00)	0.394
ASA score, n (%)			0.842			1
I	9 (9.8)	4 (13.3)		2 (7.4)	3 (11.1)	
II	77 (83.7)	24 (80.0)		23 (85.2)	23 (85.2)	
III	6 (6.5)	2 (6.7)		2 (7.4)	1 (3.7)	
Distance from AV (cm) *	3.00 (0.00, 12.00)	3.00 (0.00, 10.00)	0.039	3.00 (0.00, 10.00)	3.00 (0.00, 10.00)	0.523
Preoperative treatment, n (%)			0.206			0.779
No	51 (55.4)	12 (40.0)		9 (33.3)	11 (40.7)	
Yes	41 (44.6)	18 (60.0)		18 (66.7)	16 (59.3)	
cT, n (%) **			0.555			1
is	0 (0.0)	1 (3.3)		0 (0.0)	0 (0.0)	
1	16 (17.4)	4 (13.3)		2 (7.4)	3 (11.1)	
2	27 (29.3)	9 (30.0)		9 (33.3)	8 (29.6)	
3	39 (42.4)	14 (46.7)		13 (48.1)	14 (51.9)	
4	10 (10.9)	2 (6.7)		3 (11.1)	2 (7.4)	
cN, n (%) **			0.76			0.677
0	51 (55.4)	16 (53.3)		9 (33.3)	13 (48.1)	
1	16 (17.4)	4 (13.3)		7 (25.9)	4 (14.8)	
2	9 (9.8)	2 (6.7)		3 (11.1)	2 (7.4)	
3	16 (17.4)	8 (26.7)		8 (29.6)	8 (29.6)	
cStage, n (%) **			0.347			0.561
0	0 (0.0)	1 (3.3)		0 (0.0)	0 (0.0)	
I	37 (40.2)	10 (33.3)		7 (25.9)	8 (29.6)	
II	12 (13.0)	4 (13.3)		2 (7.4)	4 (14.8)	
III	35 (38.0)	10 (33.3)		15 (55.6)	10 (37.0)	
IV	8 (8.7)	5 (16.7)		3 (11.1)	5 (18.5)	

Abbreviations: *PSM*, propensity score matching; *BMI*, body mass index; *ASA*, American Society of Anesthesiologists; *AV*, anal verge

^*^The data are expressed as the median (range)

^**^Tumors were classified according to the American Joint Committee on Cancer (AJCC) TNM system

The surgical outcomes are shown in Table 2. The surgical procedure, lymphadenectomy, and LLND were performed approximately equally between the two groups. The median operation time, blood loss, and the number of lymph nodes harvested were also comparable between the two groups. No patients were converted to open surgery in either group.

**Table 2:** Operative outcomes after PSM

	Conventional	Hybrid	*P*
	*n* = 27	*n* = 27	
Operative procedure, n (%)			0.788
LAR	17 (63.0)	15 (55.6)	
ISR	2 (7.4)	4 (14.8)	
APR	8 (29.6)	8 (29.6)	
Lymphadenectomy, n (%) *			1
prxD1	0 (0.0)	0 (0.0)	
prxD2	1 (3.7)	2 (7.4)	
prxD3	26 (96.3)	25 (92.6)	
LLND, n (%)			1
No	15 (55.6)	15 (55.6)	
Yes	12 (44.4)	12 (44.4)	
Operation time (min) **	257.00 (179.00, 760.00)	281.00 (205.00, 579.00)	0.703
Blood loss (ml) **	0.00 (0.00, 1770.00)	0.00 (0.00, 150.00)	0.472
Transfusion, n (%)			1
No	26 (96.3)	27 (100.0)	
Yes	1 (3.7)	0 (0.0)	
Conversion, n (%)			1
No	27 (100.0)	27 (100.0)	
Yes	0 (0.0)	0 (0.0)	
Harvested LNs **	13.00 (4.00, 44.00)	15.00 (2.00, 35.00)	0.815

Abbreviations: *PSM*, propensity score matching; *LAR*, low anterior resection; *ISR*, intersphincteric resection; *APR*, abdominoperineal resection; *LLND*, lateral pelvic lymph node resection; *LN*, lymph node

^*^According to the Japanese Society for Cancer of the Colon and Rectum guidelines

^**^The data are expressed as the median (range)

The postoperative complications are summarized in Table 3. The rate of complications of grade II or higher on the Clavien–Dindo classification system was similar in both groups. The median postoperative hospital stay in the hybrid group tended to be shorter than that in the conventional group; however, no significant difference was noted between the two groups (*P* = 0.076). One case in the conventional group received re-operation for anastomotic leakage. Two and one in the hybrid group underwent re-operation due to anastomotic leakage and bowel obstruction, respectively.

**Table 3:** Postoperative outcomes after PSM

	Conventional	Hybrid	*P*
	*n* = 27	*n* = 27	
Postoperative complications (CD≧II), n (%)	16 (59.3)	11 (40.7)	0.276
Urinary disturbance	4 (14.8)	2 (7.4)	
Anastomotic leakage	1 (3.7)	2 (7.4)	
Anastomotic bleeding	0 (0.0)	0 (0.0)	
Abdominal wound infection	0 (0.0)	0 (0.0)	
Perineal wound infection	1 (3.7)	1 (3.7)	
Bowel obstruction	3 (11.1)	1 (3.7)	
Paralytic ileus	1 (3.7)	0 (0.0)	
Lymphorrhea	0 (0.0)	0 (0.0)	
Bladder injury	1 (3.7)	0 (0.0)	
Ureteral injury	0 (0.0)	0 (0.0)	
Urethral injury	0 (0.0)	0 (0.0)	
Colonic conduit prolapse	0 (0.0)	0 (0.0)	
DVT/PTE	0 (0.0)	0 (0.0)	
Pneumonia	1 (3.7)	0 (0.0)	
Others	5 (18.5)	5 (18.5)	
Postoperative complications (CD≧III), n (%)	6 (22.2)	4 (14.8)	0.728
Postoperative hospital stay (day) *	20.00 (8.00, 60.00)	16.00 (9.00, 27.00)	0.076
Re-operation ≦ 30 days, n (%)	1 (3.7)	3 (11.1)	0.610
Mortality ≦ 30 days, n (%)	0 (0.0)	0 (0.0)	1

Abbreviations: *PSM*, propensity score matching; *CD*, Clavien-Dindo classification; *DVT*, deep vein thrombosis; *PTE*, pulmonary thromboembolism

^*^The data are expressed as the median (range)

The pathological outcomes are shown in Table 4. In one case of intersphincteric resection in the hybrid group, both DM and radial margin (RM) were 450 µm, resulting in an R1 resection. However, all the other cases were negative for both DM and RM. In the conventional group, a positive RM was observed in only one case.

**Table 4:** Pathological outcomes after PSM

	Conventional	Hybrid	*P*
	*n* = 27	*n* = 27	
Histological type, n (%)			0.791
tub1/tub2	21 (77.8)	20 (74.1)	
por/sig/muc	5 (18.5)	4 (14.8)	
others	1 (3.7)	3 (11.1)	
pT, n (%) *			0.948
0	3 (11.1)	2 (7.4)	
1	4 (14.8)	6 (22.2)	
2	7 (25.9)	7 (25.9)	
3	11 (40.7)	11 (40.7)	
4	2 (7.4)	1 (3.7)	
pN, n (%) *			0.624
0	18 (66.7)	15 (55.6)	
1	6 (22.2)	8 (29.6)	
2	2 (7.4)	1 (3.7)	
3	1 (3.7)	3 (11.1)	
pStage, n (%) *			0.668
0	3 (11.1)	1 (3.7)	
I	8 (29.6)	9 (33.3)	
II	7 (25.9)	4 (14.8)	
III	6 (22.2)	8 (29.6)	
IV	3 (11.1)	5 (18.5)	
Lymphovascular invasion, n (%)			1
Absent	14 (51.9)	13 (48.1)	
Present	13 (48.1)	14 (51.9)	
DM involvement, n (%)	0 (0.0)	1 (3.7)	1
RM involvement, n (%)	1 (3.7)	1 (3.7)	1

Abbreviations: *PSM*, propensity score matching; *por*, poorly differentiated adenocarcinoma; *sig*, signet-ring cell carcinoma; *muc*, mucinous adenocarcinoma; *DM*, distal margin; *RM*, radial margin

^*^Tumors were classified according to the American Joint Committee on Cancer (AJCC) TNM system

The recurrence data are summarized in Table 5. Three patients in the conventional group and five in the hybrid group with stage IV rectal cancer were excluded, and the remaining patients were examined for recurrence. The median follow-up period was 24.0 months in the conventional group and 7.5 in the hybrid group. No significant differences were observed between the two groups concerning the recurrence, distant metastasis, and local recurrence rates.

**Table 5:** Recurrence rate for patients excluding Stage IV after PSM

	Conventional	Hybrid	*P*
	*n* = 24	*n* = 22	
Follow-up period (month) *	24.00 (6.00, 69.00)	7.50 (1.00, 24.00)	
Recurrence rate, n (%)	4 (16.7)	1 (4.5)	0.349
Local recurrence rate, n (%)	1 (4.2)	1 (4.5)	1
Distant metastasis rate, n (%)	3 (12.5)	0 (0.0)	0.235

Abbreviations: *PSM*, propensity score matching

^*^The data are expressed as the median (range)

**Fig. 2:**
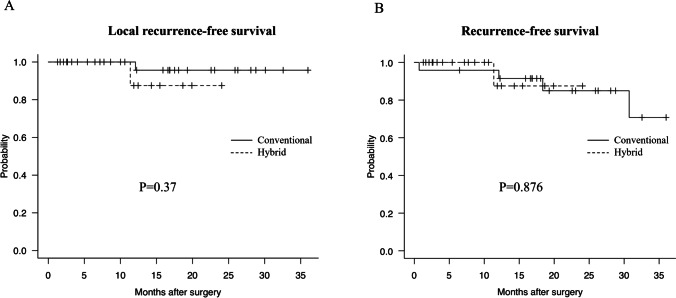
Comparison of the Kaplan–Meier curves between the conventional and hybrid transanal total mesorectal excision. **A** Local recurrence-free survival and **B** recurrence-free survival rates, excluding patients with stage IV cancer

The Kaplan–Meier curves for LRFS and RFS are shown in Fig. 2. No significant difference was found in LRFS (*P* = 0.37) and RFS (*P* = 0.876) between the two groups.

## Discussion

The application of TaTME has expanded globally since it was first described by Sylla et al. [27]. TaTME provides a better view under direct vision in low rectal cancer, secures DM and CRM, and helps preserve the pelvic nerves [16, 17]. Robotic surgery also has several important features not found in laparoscopic surgery, such as a three-dimensional stabilized camera, motion scaling, and articulating functions, allowing for more meticulous manipulation in the complex pelvis. The potential advantages of both techniques are lower rates of conversion and the consequent postoperative complications, as well as better specimen quality, leading to favorable oncologic outcomes [14, 17, 18, 28–33]. In addition, these surgical techniques are especially beneficial in patients with obesity and male patients with a narrow pelvis, which present difficulties for laparoscopy [7–9, 15, 34]. Based on the belief that incorporating the advantages of each of these techniques could achieve a safer and less invasive surgery and, consequently, better oncological outcomes, we established hybrid TaTME at our institution. To our knowledge, few reports of this technique have been published [21–24], and the present study is the first to compare it with conventional TaTME regarding safety and feasibility. We found that the short-term outcomes of hybrid TaTME were comparable to those of conventional TaTME, which was considered acceptable.

Robotic surgery has a longer operation time compared with open or laparoscopic surgery [29, 30]. Although performing hybrid TaTME would also presumably take longer in the present study, the operation time was similar to that of conventional TaTME. The operation time in the present study was also comparable to that in a previous report, although about half of the patients underwent LLND [34]. This result may be because the surgeon’s progress in robotic surgery, which enabled safer and more precise manipulation from early on, could shorten the operation time, despite the surgeon’s limited experience with robotic surgery in the present study. The operation time in hybrid surgery could also be shortened by standardizing the procedure and adhering to the time course shown in Fig. 1 through close collaboration between the two surgical teams. The accumulation of more cases is expected to eliminate the influence of the learning curve, allowing the operation time in this technique to be further shortened.

The advantages of hybrid TaTME are the improved visibility and ergonomics compared with conventional TaTME due to the various features of robotic surgery. Furthermore, the TilePro function allows the surgeon of the abdominal team to share the surgical field with the perineal team, enhancing the collaboration between both teams. These advantages may have allowed for safer and more meticulous manipulation at the various surgical stages, even in cases of a confined pelvis, ultimately leading to fewer complications. In the present study, however, while fewer complications were observed in the hybrid group than in the conventional group, no significant difference was found in the complication rate due to the small number of cases. Furthermore, the complication rate was comparable to those of previous reports, ranging from 20 to 45%, suggesting that hybrid surgery is as safe and feasible as conventional surgery [16–19, 34]. However, as this technique is still in its infancy, it is expected that further refinement through the accumulation of cases could provide more favorable short-term outcomes in complex rectal cancers.

Enhancing specimen quality to reduce local recurrence is of great importance [3–6]. While a low local recurrence rate of 0–4% has been reported for TaTME, certain concerns have been raised regarding local recurrence after TaTME, as reported in a 2019 Norwegian study, due to the complexity and difficulty of the procedure [17, 20, 31, 35]. In the hybrid group in the present study, local recurrence was observed in only one patient (4.5%), and no local recurrence was observed in patients who achieved R0 resection. These recurrence cases may be due to the positive DM or RM at the time of the initial surgery, which may not present a serious concern regarding recurrence in hybrid TaTME if the quality of the resection is maintained. In the present study, all but one patient achieved R0 resection (96.3%), and the median number of lymph nodes harvested was 12 or more in the hybrid group, which is comparable to that in the conventional group.

These pathological results were also comparable to those in previous reports on TaTME [17, 18]. Therefore, hybrid TaTME could also adequately maintain the quality of radical resection as conventional TaTME, leading to acceptable outcomes in local recurrence, despite the high proportion of cT3/4-positive, preoperatively treated, and male patients in the present study.

Another important issue is whether TaTME is still needed in the times of robotic surgery. It is well known that successful TME can be achieved by robotic surgery alone even in the obese or male patients [15]. However, TaTME is only one down-to-up approach which is completely different from the other transabdominal approaches including robotic and laparoscopic TME and should have its unique advantages. TaTME may be beneficial for the patients with bulky tumors, recurrent tumors, and positive lateral pelvic lymph nodes. Although future randomized trials of TaTME vs. robotic surgery are necessary to determine which patients benefit from TaTME, we believe that TaTME is worth learning for colorectal surgeons and that hybrid TaTME could be a useful option in the future.

This study has several limitations. First, since this is a retrospective cohort study, a bias in patient selection remains, despite performing PSM to minimize it. Second, a risk of overestimation concerning RM may exist because, in Japan, the lymph nodes in the mesorectum are generally separated from the specimen before submission to the pathology department. Finally, due to the short follow-up duration, the recurrence rate may be underestimated, thus necessitating a longer follow-up period.

In conclusion, hybrid TaTME, which incorporates the advantages of abdominal robotic surgery and TaTME, is as favorable as conventional TaTME in producing suitable short-term outcomes in low rectal cancer. Further larger-scale studies conducted over a longer period are needed to verify the current findings on this surgical technique.

## Data Availability

The data presented in this study are available on request from the corresponding author.
